# Micro and Macroscale Drivers of Nutrient Concentrations in Urban Streams in South, Central and North America

**DOI:** 10.1371/journal.pone.0162684

**Published:** 2016-09-23

**Authors:** Steven A. Loiselle, Davi Gasparini Fernandes Cunha, Scott Shupe, Elsa Valiente, Luciana Rocha, Eleanore Heasley, Patricia Pérez Belmont, Avinoam Baruch

**Affiliations:** 1 Earthwatch Institute, Oxford, United Kingdom; 2 Departamento de Hidráulica e Saneamento, Escola de Engenharia de São Carlos, Universidade de São Paulo, São Carlos, SP, Brasil; 3 Geography and the Environment, University of the Fraser Valley, Abbotsford, BC, Canada; 4 Restauración Ecológica y Desarrollo A.C., Ciudad de México, México; 5 Grupo de Ecología Acuática, Instituto de Ecología y Desarrollo Sustentable (INEDES), CONICET y Departamento de Ciencias Básicas, Universidad Nacional de Luján, Luján, Buenos Aires, Argentina; 6 Department of Geography, Loughborough University, Loughborough, Leicestershire, United Kingdom; University of Arkansas Fayetteville, UNITED STATES

## Abstract

Global metrics of land cover and land use provide a fundamental basis to examine the spatial variability of human-induced impacts on freshwater ecosystems. However, microscale processes and site specific conditions related to bank vegetation, pollution sources, adjacent land use and water uses can have important influences on ecosystem conditions, in particular in smaller tributary rivers. Compared to larger order rivers, these low-order streams and rivers are more numerous, yet often under-monitored. The present study explored the relationship of nutrient concentrations in 150 streams in 57 hydrological basins in South, Central and North America (Buenos Aires, Curitiba, São Paulo, Rio de Janeiro, Mexico City and Vancouver) with macroscale information available from global datasets and microscale data acquired by trained citizen scientists. Average sub-basin phosphate (P-PO_4_) concentrations were found to be well correlated with sub-basin attributes on both macro and microscales, while the relationships between sub-basin attributes and nitrate (N-NO_3_) concentrations were limited. A phosphate threshold for eutrophic conditions (>0.1 mg L^-1^ P-PO_4_) was exceeded in basins where microscale point source discharge points (eg. residential, industrial, urban/road) were identified in more than 86% of stream reaches monitored by citizen scientists. The presence of bankside vegetation covaried (rho = –0.53) with lower phosphate concentrations in the ecosystems studied. Macroscale information on nutrient loading allowed for a strong separation between basins with and without eutrophic conditions. Most importantly, the combination of macroscale and microscale information acquired increased our ability to explain sub-basin variability of P-PO_4_ concentrations. The identification of microscale point sources and bank vegetation conditions by citizen scientists provided important information that local authorities could use to improve their management of lower order river ecosystems.

## Introduction

Anthropogenic stressors endanger more than 65% of fluvial habitats globally [1]. Increased nutrient loads and reduced ecosystem functioning have led to algal blooms and widespread artificial eutrophication in most freshwater ecosystems. This is evident in both periurban and rural ecosystems, where land management has a strong influence on nutrient fluxes, in comparison to the dominant climate influences in undisturbed areas [2, 3]. In urban and periurban areas, elevated impervious land cover modifies nutrient dynamics [4–6] and particulate inputs [7]. In agriculturally dominated areas, increasingly industrial-scale activities utilise major inputs of mineral based nutrients which have basin-scale (and long term) impacts on the nutrient dynamics of rivers, river sediments and receiving waterbodies [8]. The resulting eutrophication modifies macroinvertebrate and native fish populations, carbon sequestration and in-stream vegetation diversity (eg. favouring harmful algal blooms), effectively changing the basis of ecosystem functioning [9–12].

The use of satellite based estimates of land cover and land use provides a fundamental basis to understand the spatial variability of human-induced impacts on freshwater ecosystems [13]. However, microscale processes and site specific conditions related to bank vegetation, pollution sources, adjacent land and water use have been shown to impact biological communities [14–16]. While macroscale information from Earth Observation (land cover/use) is increasingly available, microscale data require local data gathering. Acquisition of such high resolution field data is resource (cost, time) intensive. Most monitoring programmes focus on a limited number of typically large waterbodies (i.e., usually the most important tributaries of a given catchment area). This is particularly problematic as the majority of water bodies are small and therefore unmonitored [17].

Clearly, there is a need for new data acquisition approaches. One possible source of additional data is that acquired by trained citizen scientists–non-professional scientists or volunteers with basic training in data collection and ecosystem analysis. Citizen science is increasingly being relied on to improve the temporal and spatial resolution of local data acquisition, complementary to agency monitoring programmes [18–20]. This approach depends on appropriate training [21] and the presence of a local community willing to collaborate.

Combining macroscale and microscale information gathered through citizen scientists represents a novel opportunity to identify the conditions of freshwater ecosystems and the factors which influence their degradation. However, the relative importance of microscale data with respect to larger macroscale information for explaining ecosystem conditions remains unclear. This has important consequences as microscale conditions are more amenable to management actions (restoration, mitigation) than macroscale land use changes. The determination of threshold values for microscale conditions would allow more effective decision making.

In the present study, we explored the use of high resolution microscale data gathered by citizen scientists to improve the explanatory power of low resolution macroscale information on river stressors in stream basins in South, Central and North America. We hypothesised that nutrient concentrations are sensitive to potential drivers at both macro and microscales and that the latter are complementary to the former. To our knowledge, this is the first study to associate data acquired by citizen scientists with macroscale information for the analysis of freshwater ecosystems.

## Methods

Our analysis was based on the hypothesis that information at two very different scales, macroscale data (globally available) and microscale data (obtained by local citizen scientists) would provide insights to different nutrient pathways and processes, allowing for a more robust analysis of sub-basin conditions [22, 23]. We focused on microscale data that would best describe point sources and processes (pollution sources, bankside vegetation, local land use/cover) and macroscale data for diffuse processes and sources (nutrient loadings, general land use/cover and population). Additional hydrological variables (stream order, sub-basin size and sampling day precipitation) were also expected to influence nutrient conditions [24].

### Macroscale data acquisition

Drainage basin boundaries (HydroBasins) for each study area were extracted from the 15 arc-second resolution USGS HydroSHEDS database. HydroBasins are nested hierarchically into 12 levels following the Pfafstetter coding system [25]. For this study, level 10 basins were used, which provided an appropriate scale for aggregating samples into similar sized basins with common geological and climate conditions. Stream order (Strahler classification) was determined using 15 arc-second resolution USGS HydroSHEDS flow accumulation and flow direction data, using a minimum accumulation condition of 100 cells. Population densities for 2010 were obtained from the Columbia University Center for International Earth Science Information Network [26, 27]. Daily precipitation data was obtained from the Global Precipitation Climatology Project [28]. The Adjusted Human Water Security (AHWS) index was used as well as its component datasets (30' resolution latitude x longitude) for nutrient loading and land cover fractions for the year 2000 [29]. The AHWS combines key global drivers regarding water resource development (human and agricultural), pollution (nutrient loading), watershed disturbances (cropland and livestock density) and biotic factors (fishing and invasive species).

### Field measurements

**Fig 1:**
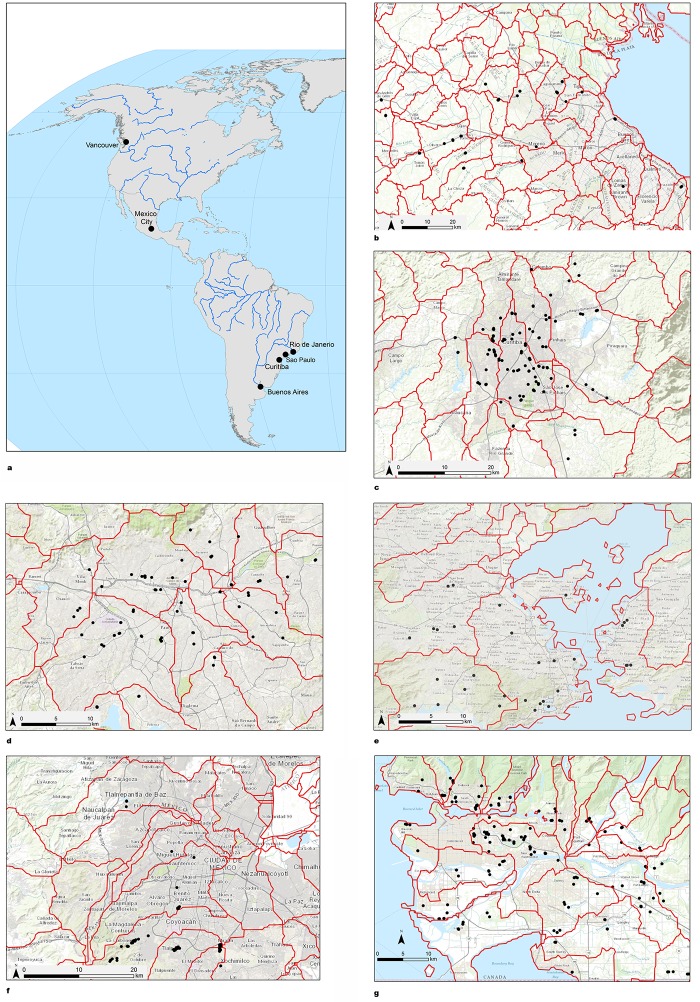
a) Global location of study areas, b) Sub-basins and sampling sites examined by citizen scientists in Buenos Aires, c) Curitiba, d) São Paulo, e) Rio de Janeiro, f) Mexico City and g) Vancouver. Basemap image reprinted from Esri under a CC BY license with permission from Esri and its licensors, original copyright June 2009.

Between September 2013 and September 2015, 1,000 trained citizen scientists, working in groups of 2 or 3, collected 2,097 datasets from 150 rivers and streams in urban and periurban areas in Buenos Aires, Curitiba, São Paulo, Rio de Janeiro, Mexico City and Vancouver as part of the FreshWater Watch programme. Measurements were repeated bimonthly or quarterly in the same sample sites, assigned by project scientists to cover urban, periurban and quasi-rural streams that were not being monitored by local authorities. Additional sites (13%) were self-selected by participants. The locations and measurements obtained at each sample are available at: freshwaterwatch.thewaterhub.org/content/data-map and the site locations are presented in Fig 1.

Each dataset contained observations and measurements of ecosystem conditions, hydrology and water quality, collected using a consistent methodology and uploaded directly on to the online database available. General ecosystem conditions included observations of the land use/cover in the immediate surroundings of the sampling site, visible evidence of pollution sources (e.g. discharge pipes) and estimates of their potential sources (urban or road runoff/drainage, residential, industrial, other) and the presence of bankside vegetation at the sampling point. These observations were limited to the immediate area of the sampling site, in general less than 25 m in both directions. Hydrological conditions were assessed using categorical estimates of water flow. Menu-based observations of water colour, the presence of pollution features (oil, foam, litter) and algal blooms were also recorded for each site and supported by a photographic documentation [19].

Measurements of dissolved phosphate (P-PO_4_) and nitrate (N-NO_3_) concentrations were performed from unfiltered samples using colorimetric methods. The method allowed for in-situ estimates of dissolved nutrients with exposure to reagents occurring within closed sample tubes, a method appropriate for a mass citizen science programme. Total nutrient concentrations could not be measured in the field by citizen scientists due to digestion and laboratory analysis requirements. Phosphate concentrations were estimated using inosine enzymatic reactions in seven specific ranges from 0.02 mg L^-1^ to 1.0 mg L^-1^ P-PO_4_ [30, 31]. Nitrate concentrations were measured using N-(1-napthyl)-ethylenediamine [32] in seven specific ranges from 0.2 mg L^-1^ to 10 mg L^-1^ N-NO_3_.

Field methods were tested against laboratory methods [33] and calibrated sensors using standard solutions and natural water samples. Duplicate and triplicate measurements were made during training and quarterly quality control analysis. Variability between different citizen scientists in the same waterbodies (on the training days) was assessed. All data were cross-checked against specified criteria. If an inconsistent measurement was found, the citizen scientist who collected the dataset was notified and asked to confirm, delete or correct the measurement.

Datasets were time and geo-coded either using a dedicated smartphone app or online using measured geographic coordinates or Google maps. After uploaded to a common database, all data were checked by project scientists. All participants were trained to use consistent data acquisition methods by professional scientists in field-based training days and were required to pass an online training quiz before being able to upload data. Written instruction sheets were provided with each testing kit and a training video was used to remind participants of the appropriate methods.

### Data analysis

Phosphate and nitrate concentrations were averaged within individual sub-basins (L10 HydroBasin) to determine a sub-basin average. Of the 97 original sub-basins, only those with more than 10 measurements were used for the analysis (n = 57). The average number of measurements was 34 per sub-basin, covering typically quarterly measurements of 3 streams per basin, with an average sub-basin area of 146 km^2^. The experimental unit of all further analysis is that of the sub-basins.

Microscale information on the number of pollution sources (eg. industrial, residential, road discharge) observed and recorded by the citizen scientists during each measurement was summed to create an index of point pollution sources for each sampling site. Observations of site-adjacent land use/cover were recorded individually and aggregated into three categories based on assumed potential impact (0-forest, 1-urban park, grassland/pasture 2-agricultural, industrial, and/or urban residential). Observations of site specific bank vegetation were divided into vegetated (1) and non-vegetated values (0). All values were averaged across sub-basins.

Macroscale and microscale sub-basin averages were compared to nutrient concentrations using non-parametric tests (Mann-Whitney U, Spearman’s rank correlation). Correlations above 0.6 were considered strong following Tukey’s guidelines and multiple hypotheses corrections (Bonferroni) for significance were utilised.

Receiver operating characteristic (ROC) analysis was used to identify possible thresholds for macroscale and microscale characteristics with respect to elevated nutrient concentrations [34, 35]. ROC analysis is commonly used to understand the performance of a binary classifier [36], in this case, sub-basins with eutrophic conditions based on P-PO_4_ concentrations. A single concentration limit for eutrophication is difficult to determine and will depend on the local geological, climate and groundwater conditions [37]. Nevertheless, we used a P-PO_4_ concentration of 0.1 mg L^-1^ for rivers and streams to discriminate sub-basins with eutrophic conditions [38, 39]. We calculated area under the ROC curve to compare the explanatory characteristics (specificity and sensitivity) of individual microscale and macroscale variables using SPSS (version 21). Only those variables that were found to have statistically significant rho were used in the ROC analysis, considering a Bonferroni-corrected significance of 0.002 (0.05/24).

Nutrient concentrations were log transformed for multiple linear regression analysis (kurtosis & skewness outside the range of –1.0 to 1.0). Multiple linear regression (backward step regression, removal criteria for probability of F < 0.05.) with SPSS (version 21) was used to identify which microscale and macroscale variables, or combination of variables, contribute to elevated P-PO_4_ and N-NO_3_ concentrations. Those variables that were found to have statistically significant rho, corrected for multiple hypotheses, were used in the regression analysis. Multicollinearity of variables was identified using a Variance Inflation Factor above 2.5 and noted in the results. Partial correlations and homoscedasticity were also checked. Models were evaluated based on the highest adjusted R^2^.

## Results

**Fig 2:**
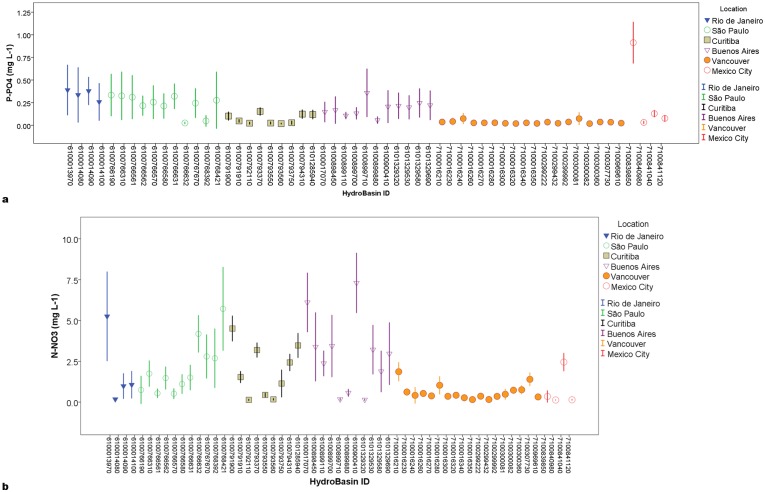
Average concentrations of phosphate (a) and nitrate (b) measured in each sampled sub-basins (Level 10 HydroBasins on y axis) in Buenos Aires, Curitiba, Mexico City, Rio de Janeiro, São Paulo and Vancouver. Bars indicate 2 standard errors (SE).

Nutrient concentrations varied greatly across sub-basins and cities (Fig 2, S1 Table), with the highest concentrations (means and medians) in sub-basins in Mexico City, Rio de Janeiro and São Paulo, and the lowest in Curitiba and Vancouver.

Sub-basins had differences in land cover, from highly urban to rural with a very low population density. São Paulo had the largest coverage of impermeable surfaces and population density (S1 Table). AHWS was highest in Rio de Janeiro, São Paulo and Mexico. Nutrient loading was greatest in São Paulo and livestock density was most elevated in Curitiba.

Average stream order was 1.6 (±0.8) with sub-basins in São Paulo having the highest stream order. Average rainfall on the day of sampling was 3.5 (± 3.1) mm/day with sub-basins in Curitiba having the highest average daily rainfall. Sub-basin areas averaged 146 (± 77) km^2^ with the smallest basins in São Paulo and the largest in Rio de Janeiro.

**Fig 3:**
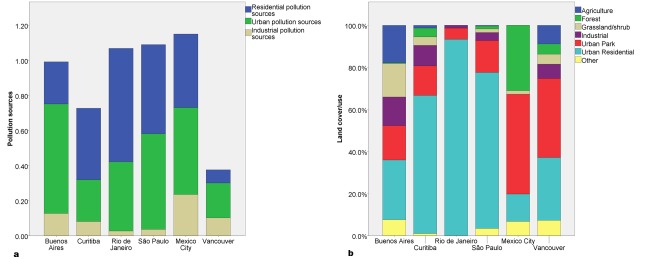
(a) Microscale pollution sources identified by citizen scientists reported as the average sum of each pollution source category per sub-basin averaged by city and (b) percentage of land cover/use recorded by citizen scientists per sub-basin averaged by city.

On a microscale, the average number of observed site-specific pollution sources (discharges) was 0.8 per measurement site (S1 Table), with residential and urban/road sources being most often identified (Fig 3a). Vegetated stream banks were observed in 93% of the sampling sites, and microscale land cover was mostly urban residential followed by urban park (Fig 3b).

High correlations (rho > 0.6, n = 57) between basin averaged phosphate concentrations and nutrient loading (and organic matter) were observed (Table 1). Interestingly, the observed sum of microscale pollution sources was the best covariate of phosphate concentration, with a correlation coefficient of 0.70. Moderate correlations (rho from 0.4 to 0.6) of phosphate concentrations with macroscale characteristics of impervious land cover, AHWS and population density were found. Moderate correlations with microscale variables (bank vegetation cover and land use/cover) were also evident (Table 1).

**Table 1:** Spearman’s rho between sub-basin averaged phosphate and nitrate concentrations (n = 57) and microscale and macroscale variables. *AHWS refers to the Adjusted Human Water Security, [29]. **Significant p-values, considering multiple hypotheses are below 0.002 (Bonferroni correction).

	Phosphate		Nitrate	
	Spearman's rho	p-value**	Spearman's rho	p-value
**Microscale data (observed by participants)**				
Land use/cover impact category	0.499	<0.001	0.365	0.005
Sum of pollution sources	0.699	<0.001	0.264	0.047
Stream bank vegetation	–0.534	<0.001	–0.139	0.301
**Hydrological data**				
Sub-basin area (km^2^)	0.117	0.381	–0.13	0.334
Precipitation	–0.144	0.286	0.177	0.188
Stream Order	–0.166	0.216	0.094	0.485
**Macroscale data**				
Cropland land cover fraction	–0.358	0.01	–0.418	0.001
Impervious land cover fraction	0.521	<0.001	0.366	0.005
Nitrogen loading	0.625	<0.001	0.306	0.021
Phosphorus loading	0.639	<0.001	0.313	0.018
AHWS*	0.518	<0.001	0.136	0.313
Population density	0.472	<0.001	0.415	0.001

Macro and microscale influences on nitrate concentrations were lower, with no high correlations and limited variables with moderate correlations. These were limited to two macroscale variables: cropland land cover fraction (moderate) and population density (low).

**Fig 4:**
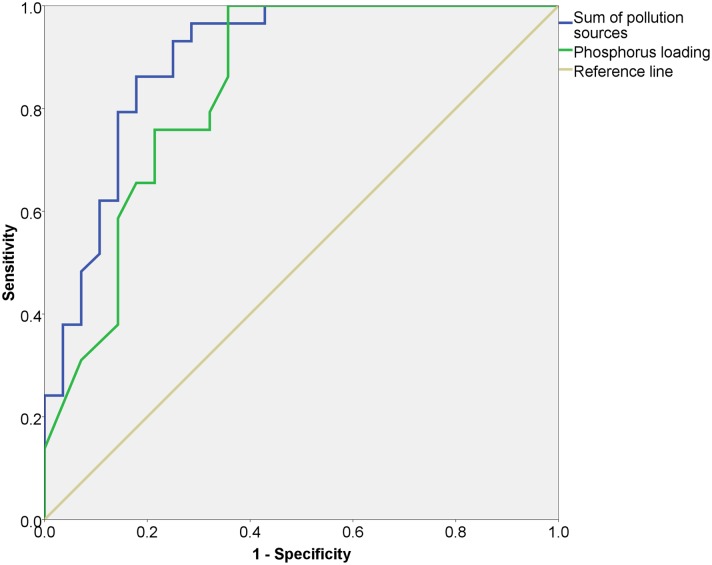
Receiver Operating Characteristic curve for the phosphate concentration eutrophication threshold of 0.1 mg L^-1^ for sub-basins against microscale observations of the sum of pollution sources and macroscale data related to phosphorus loading. The x-axis is denominated as 1 minus Specificity or the false positive rate.

Receiver operating characteristic (ROC) analysis for a phosphate concentration limit of 0.1 mg L^-1^ suggested that macroscale characteristics (phosphorus loading) and microscale data (sum of pollution sources) provided significant estimates (p<0.01) with greater than 0.80 area under the ROC curve. The sum of pollution sources provided a slightly higher area under the curve (0.89) with respect to phosphorus loading (0.84), where a perfect classifier has an area of 1 (percentage of total area) and a poor classifier has an area of 0.50 [35]. The determination of area was insensitive to the relative distribution of the two classes, eutrophic and non-eutrophic. Using a value of 0.75 for sensitivity (true positive rate) and 0.25 (1–0.75) for specificity (false positive rate), the threshold for phosphate loading was 0.975 (Fig 4). Using a value of 0.75 for sensitivity (true positive rate) and 0.15 (1–0.85) for specificity (false positive rate), the threshold for the sum of local pollution sources was 0.855.

The macro and microscale data, combined using multiple linear regression, did not show a strong relationship with nitrate (log10 transformed to reduce skewness). Phosphate concentrations (log10 transformed to reduce skewness) showed a stronger relationship with both macroscale and microscale data (Table 2). Considering the former, a combination of macroscale phosphate loading (Phosphate loading) estimates and AHWS provided the best model, with a moderate correlation in explaining the variability of phosphate concentrations in each sub-basin (adjusted R^2^ of 0.31, Table 2). The results of the model showed some collinearity as tolerance was 0.251 and the distribution of the standardised residuals was skewed towards higher predicted values (signs of heteroscedasticity). Partial correlations showed that the relationships between each variable and P-PO_4_ concentrations (log transformed) remained significant, controlling for the effects of the other variable. The use of only microscale observations (sum of pollution sources and bank vegetation) provided better explanatory power, reaching an adjusted R^2^ of 0.47, with a much higher tolerance of 0.847, a scatter plot of standardised residuals showing no trend and partial correlations remaining significant.

**Table 2:** Multiple linear regression analysis for sub-basin phosphate concentrations using microscale and macroscale variables.

Model Predictors	R	R Square	Adjusted R Square	Std. Error	Durbin-Watson
Phosphorus loading, AHWS, Bank vegetation, Sum of pollution sources	.72	.51	.48	.256	1.906
Bank vegetation, Sum of pollution sources	.70	.49	.47	.258	1.813
Phosphorus loading, AHWS	.58	.34	.31	.293	1.740

Combining both microscale and macroscale information allowed for a large improvement over the use of macroscale variables alone, and a small improvement over using microscale variables alone (Table 2). The resulting models showed low collinearity between parameters (tolerance between 0.8 and 0.9) and there was no serial correlation among the residuals (Durbin-Watson). Cooks distances were all below 0.2 and the scatterplot of standardised residuals showed no trends.

## Discussion

Nitrate concentrations were not well correlated to either macroscale or microscale land cover/use variables using the sub-basin averages in the six study areas. Previous studies show correlations between nitrate and agriculture land cover [40, 41] for large river networks. The weaker correlations in our study for nitrate compared to phosphate may have resulted from the more dynamic nature of nitrate cycling (with respect to phosphate) in small waterbodies. The study areas covered a range of climate conditions, with large differences in temperature, residence time, oxygen conditions, groundwater inputs and surface temperatures, all with important impacts on dissolved nitrate dynamics [42–44]. As these variables were not evaluated in the present study, key drivers of nitrate dynamics were left out of the present analysis.

Phosphate concentrations showed important correlations to both macroscale and microscale variables. The positive relationship between phosphate concentrations and macroscale descriptors, based on low resolution global land cover data, confirmed the usefulness of satellite based land cover data to study aquatic systems conditions. These globally available data allowed for a good estimate of the variability of phosphate concentrations across a range of river environment and climate conditions. These databases, and in particular AHWS, were developed to examine broad patterns of water quality for large river networks (stream order > 5, [29]). It is interesting that they were successful when focused on relatively low stream order systems. It is expected that higher resolution, more current land cover datasets would provide better results. Such information, if available on a global scale, would greatly improve our capacity to explore basin scale impacts on freshwater ecosystems across biomes. At present, most large scale studies are limited to temperate areas [9].

Average sub-basin phosphate concentrations ranged from 0.39 mg L^-1^ in one sub-basin in Rio de Janeiro to 0.018 mg L^-1^ in sub-basins in Curitiba and Vancouver, with an average of 0.15 mg L^-1^. This matches well with the average P-PO_4_ concentration for all river and streams in the 30 cities of FreshWater Watch: 0.15 mg L^-1^ from August 2013 to April 2016 (n = 7,646). It also matches well with the average P-PO_4_ concentration reported for all surface waters (including lakes) in the EPA NRSA/Storet database (n = 105,347, United States only, data from 1992 to 2009); 0.11 mg L^-1^. Therefore, the nutrient concentrations across our study streams spanned the range reported in existing international datasets, suggesting our findings are applicable to urban and periurban aquatic systems globally.

Considering phosphate as the main driver of eutrophication within the sub-basins, a macroscale phosphate loading threshold of 0.975 (standardised units) was shown by ROC analysis to provide a good separation of basins with more eutrophic conditions. This indicates that basins with a loading above 0.975 were correctly identified as eutrophic (exceeding 0.1 mg L^-1^ P-PO_4_) 75% of the time, and incorrectly identified as being below the P-PO_4_ limit only 25% of the time. Of the 57 sub-basins analysed, 29 (51%) had an average phosphate loading below this threshold. These were present in Buenos Aires, Curitiba and Vancouver. No threshold for the AHWS index could be identified that provided both acceptable specificity and sensitivity. Combining both phosphate loading and AHWS, regression analysis showed that 31% of the variability of the phosphate concentrations could be explained. Phosphorus loading was the most important variable, as seen by both the standardised coefficients and partial correlations. It should be noted that macroscale land use/cover data showed an elevated covariance (eg. partial correlations), a natural consequence of the gradients considered in the AHWS analysis and the link between anthropogenic and natural landscape gradients [45].

Microscale data significantly improved our capacity to explain the variance in phosphate concentrations across sub-basins, taken separately as well as in combination with macroscale data. As an individual microscale variable, the observed number of pollution sources provided the most explanatory power, while information on bankside vegetation was also found to provide moderate correlation. This supports studies regarding the importance of reducing residential discharges and fertiliser use in controlling stream nutrient conditions [46, 47, 41]. Using a phosphate concentration limit of 0.1 mg L^-1^, a microscale pollution source threshold (sum of pollution sources) of 0.855 allowed for a statistically significant separation of basins with more eutrophic conditions. This threshold was surpassed in 64%, 56%, 100%, 100%, 91% and 5%, of the sub-basins in Buenos Aires, Curitiba, Mexico City, Rio de Janeiro, São Paulo and Vancouver respectively. Effectively, this means that the observation of a pollution source near 86% of the sampling sites in a sub-basin was sufficient to accurately classify that sub-basin as eutrophic (> 0.1 P-PO_4_ mg L^-1^).

The resulting threshold indicates the importance of (typically) under-monitored and unidentified discharges in lower order rivers. Residential discharges (outfalls) and urban discharges were the most common in the study sub-basins (Fig 3a). There are few studies addressing the impact of residential land use near streams and rivers, and those that do (eg. [48]) are limited to modern residential developments where these discharges are less common. The identification of microscale point sources by trained local community members improves stakeholder capacity to explain the spatial variability of algal blooms and other impacts of eutrophication. Furthermore, this information provides stakeholders with opportunities to address local (and more manageable) drivers of ecosystem degradation. Experiments using trained community members to monitoring outfalls are underway in several areas in the UK (eg. Thames 21 [49]).

The negative relationship between bank vegetation and phosphate concentrations indicated that rivers and streams in the study areas with vegetated buffers had lower phosphate concentrations. These data did not allow for the determination of the buffering capacity of vegetated banks (no significant ROC threshold), but do lend weight to the role of vegetated buffer strips in reducing surface and subsurface inputs of nutrients into streams [50–52]. The importance of bank vegetation was less than that of pollution sources, from standardised coefficients and partial correlations, but still significant in explaining phosphate concentrations. The regression with both microscale variables explained nearly half of the variability in phosphate concentrations.

The observation of microscale land use/cover in the immediate sampling area provided limited interpretative power, indicating that microscale information is less important than macroscale land use/cover conditions in the study areas. This was demonstrated by a lower rho with respect to macroscale attributes (eg. impervious land cover fraction) and no statistically significant relationship between the microscale land use/cover parameter and nutrient concentrations using the ROC analysis. This result confirmed studies that show that macroscale land cover information provides better explanatory information in heavily modified areas with limited spatial diversity [53]. In undisturbed sub-basins, we would expect that stream nutrient concentrations may be more sensitive to microscale land use/cover differences [53].

Adding microscale information improved our overall understanding of the variability of phosphate concentrations compared to using macroscale information alone, with an increase in the adjusted R^2^ from 0.34 to 0.51, with a 13% reduction in the standard error (Table 2). All variables appear to have a similar importance in the regression equation (based on standardized regression coefficients). The tolerance of the macroscale phosphorus loading and AHWS confirmed the expected correlation between these variables. Integrating information from these two sources lends weight to ongoing studies of microscale data to model river water quality [54]. The improvement made by introducing macroscale variables to explain the variability in sub-basin phosphate conditions was limited (2% improvement in adjusted R^2^), and while topographic factors (not explored here) have been shown to be important on a microscale [55], we show that observational microscale variables provide important tools to identify nutrient conditions.

A number of macroscale variables underperformed with respect to their expected importance. Meteorology typically plays an important role in modifying nutrient concentrations. However, sampling day precipitation was not found to influence the variability of average phosphate concentrations. This may have resulted from the variable lag times for precipitation and runoff in the range of streams examined and the low resolution of the precipitation data (1.0° x 1.0°). It should also be noted that citizen acquired data may contain a bias towards sampling in non-rain conditions (for comfort and safety considerations). Interestingly, the average daily rainfall on sampling days was similar to the average daily rainfall for each study area (except for São Paulo), indicating that this bias was relatively low. However, a bias towards a reduced frequency sampling during heavy rain events is inherent in citizen based data acquisition in rivers and streams. It should be noted that for studies on nutrient dynamics, it would be advisable to use consistent lag times between rain events and data acquisition by citizen scientists with respect to whether first flush or base flow conditions are desired.

Interestingly, average stream order was not found to be an important driver of nutrient concentrations. This may be due to the similarity between the sub-basins examined, with mean stream order below 2, except Rio de Janeiro. Finally, sub-basin area did not significantly influence nutrient concentrations, contrary to studies which show the importance of basin area on nutrient concentrations [56, 22]. As most study streams were ungauged, it was not possible to normalise measurements using stream discharge or base flow conditions.

The present study focused on the use of repeated “spot” measurements of dissolved nutrient concentrations to explore the spatial variability of river basin conditions. We recognise that continuous or integrated measurements of nutrient concentrations or their impact would provide better information on nutrient dynamics. Biotic measurements and in-stream sensors provide more complete information, but may not always be appropriate for mass citizen science based measurements due to elevated cost (sensors) and time/training requirements (biological measurements) compared to grab samples or spot measurements [57]. These measurement approaches (biotic, sensor and bio-optical/chemical) are complementary, allowing for a range of participation (and training requirements).

## Conclusions

Eutrophication of surface waterbodies presents an important challenge to decision makers, which is compounded by insufficient information on ecosystem conditions and potential drivers. In the present study, we used information gathered by thousands of trained citizen scientists to explore the spatial variability of nutrient concentrations and microscale conditions of stream basins. Combining macroscale and microscale data increased our capacity to explain the variability of phosphate concentrations on a sub-basin scale. Integrating information acquired by trained citizen scientists with global datasets of land use/cover represents a new approach to explore factors that control water quality and is a key step towards managing the drivers of its degradation.

Macroscale national and international data help broadly define conditions across basins and can identify potential tipping points. In turn, microscale information is important for evaluating potential point sources of pollution and the presence of riparian buffer areas to mitigate non-point source runoff. The participation of active and informed citizens allows for a greater temporal frequency of data acquisition, while also allowing for rapid identification of changes in ecosystems before they expand into more widespread impacts. Thresholds for microscale point sources (eg., discharges) can be used in the design of early alert systems and long term monitoring processes. The identification of microscale point sources and the condition of bank vegetation provides stakeholders and local authorities with high resolution information to improve control of key drivers of ecosystem degradation.

The present study focused on predominantly smaller rivers and streams which were previously unmonitored. This is a consistent pattern internationally as smaller order streams, greater in number and in length [58] than larger rivers, are not regularly monitored. There is a clear need to increase data gathering in these spatially disperse ecosystems and the use of trained citizen scientists is one promising method to generate complementary data to government agency monitoring schemes. Microscale conditions are likely to have a larger influence on the conditions of smaller ecosystems, with respect to their larger counterparts [59, 60]. This is further justification of the integration of community based monitoring within sub-basin scale programmes. In this study, field observations and measurements made by citizen scientists were found to provide complementary information to coarser scale global data in showing patterns of water quality across a range of climate and ecological conditions.

## Supporting Information

S1 Table Average and standard deviation of the study sub-basin characteristics by city (see Methods for data sources, AHWS refers to the Adjusted Human Water Security, values missing standard deviation indicate that all sub-basin values of land cover were equal). docx
