# Supplementary material for: Micro and Macroscale Drivers of Nutrient Concentrations in Urban Streams in South, Central and North America
Source: PLoS One. 2016 Sep 23;11(9):e0162684. doi: 10.1371/journal.pone.0162684 (PMC5035044; doi:10.1371/journal.pone.0162684)
Supplement: S1 Table — docx [file pone.0162684.s001.docx]

|  | **Buenos Aires** | | **Curitiba** | | **Rio de Janeiro** | | **São Paulo** | | **Mexico City** | | **Vancouver** | |
| --- | --- | --- | --- | --- | --- | --- | --- | --- | --- | --- | --- | --- |
| **N** | 11 | | 9 | | 4 | | 11 | | 4 | | 17 | |
| **N-NO_3_**  **(mg L^-1^)** | 2.9 | ±2.3 | 1.9 | ±1.6 | 0.8 | ±1.1 | 1.9 | ±2.3 | 2.1 | ±1.6 | 0.6 | ±0.4 |
| **P-PO_4_**  **(mg L^-1^)** | 0.19 | ±0.08 | 0.07 | ±0.05 | 0.31 | ±0.47 | 0.34 | ±0.06 | 0.23 | ±0.11 | 0.03 | ±0.02 |
| **Sum of pollution sources** | 0.99 | ±0.25 | .73 | ±0.24 | 1.15 | ±0.35 | 1.07 | ±0.18 | 1.09 | ±0.34 | 0.38 | ±0.22 |
| **Bank vegetation** | 0.99 | ±0.02 | .99 | ±0.01 | 0.88 | ±0.14 | 0.77 | ±0.12 | 0.79 | ±0.27 | 0.99 | ±0.02 |
| **Cropland land cover** | 0.46 | ±0.25 | 0.55 | ±0.12 | 0.62 |  | 0.23 |  | 0.11 | ±0.09 | 0.45 | ±0.18 |
| **Impervious land cover** | 0.95 | ±0.07 | 0.97 | ±0.02 | 1.00 |  | 1.00 |  | 1.00 | ±0.00 | 37.69 | ±  155.7 |
| **Nitrogen loading** | 0.70 | ±0.15 | 0.73 | ±0.02 | 0.93 |  | 0.97 |  | 0.90 | ±0.04 | 0.34 | ±0.11 |
| **Phosphorus loading** | 0.88 | ±0.08 | 0.98 | ±0.01 | 1.00 |  | 1.00 |  | 1.00 | ±0.01 | 0.61 | ±0.14 |
| **AHWS** | 0.77 | ±0.20 | 0.96 | ±0.01 | 0.99 |  | 1.00 |  | 0.99 | ±0.00 | 0.64 | ±0.17 |
| **Population density (pp/km2)** | 3014 | ±  4552 | 2380 | ±  1799 | 4725 | ±  2962 | 7782 | ±  3679 | 9585 | ±  6820 | 1193 | ±  920 |
| **Precipitation (10x mm/day)** | 34 | ±19 | 75 | ±33 | 20 | ±21 | 9 | ±7 | 39 | ±19 | 22 | ±12 |
| **Stream Order** | 1.7 | ±1.0 | 1.6 | ±0.8 | 1.3 | ±0.4 | 1.2 | ±0.3 | 2.4 | ±1.2 | 1.7 | ±0.7 |
| **Sub-basin area (km^2^)** | 166 | ±57 | 184 | ±110 | 165 | ±41 | 246 | ±5 | 93 | ±39 | 113 | ±72 |

S1 Table. Average and standard deviation of the study sub-basin characteristics by city (see Methods for data sources, AHWS refers to the Adjusted Human Water Security, values missing standard deviation indicate that all sub-basin values of land cover were equal).
